# Synthesis of Poly(Hexamethylene Succinate-Co-Ethylene Succinate) Copolymers With Different Physical Properties and Enzymatic Hydrolyzability by Regulating the Ratio of Monomer

**DOI:** 10.3389/fbioe.2022.894046

**Published:** 2022-04-28

**Authors:** Menglu Li, Jing Jing, Tingting Su

**Affiliations:** School of Petrochemical Engineering, Liaoning Petrochemical University, Fushun, China

**Keywords:** aliphatic polyester, poly(hexamethylene succinate-co-ethylene succinate), enzymatic hydrolysis, cutinase, physical properties

## Abstract

Poly(hexylene succinate) (PHS), poly(ethylene succinate) (PES), and their random copolyesters, poly(hexylene succinate-co-ethylene succinate) ((P(HS-co-ES)), were synthesized by melting polycondensation. Simply varying the ratios of HS/ES afforded control over the copolymer crystallinity, thermal and mechanical properties, wettability, and enzymatic hydrolyzability as shown by X-ray diffraction (XRD), differential scanning calorimetry (DSC), tensile tests, and water contact angle (WCA) measurements. The enzymatic hydrolysis rates of all prepared copolyesters were higher than those of the corresponding homopolyesters. The hydrolysis rates were affected by crystallinity, melting temperature, and hydrophobicity of the copolyesters, and therefore, the degradation rates could be tuned along with the ES content. The library of copolymers prepared here with tunable degradation rates, ranging from HS-enriched to ES-enriched copolyesters, is promising for a variety of different applications. The P(HS-co-ES51) copolyester that did not fully degrade is particularly promising for use in long-term storage applications, whereas P(HS-co-ES13) and P(HS-co-ES76) that rapidly degrade are good for use in very short-term applications.

## Introduction

Although the polymer-based products are widely used, traditional polymers do not easily degrade and are a major source of pollution in soil, air, and water (Papageorgiou et al., 2014; Balart et al., 2020; Blanco et al., 2020; de Araújo Veloso et al., 2021). Degradable polymers as potential green material candidates for use in such applications have received extensive attention. One promising class of degradable polymers is aliphatic polyesters which are biocompatible, biodegradable, and thermally stable. As such, copolyesters have attracted growing attention and are used in a wide range of applications, including biomedical materials, disposable packaging, and agricultural films (Pan et al., 2018; Polyák et al., 2018; Siracusa and Blanco, 2020; Kesavan et al., 2021).

Both poly(hexylene succinate) (PHS) and poly(ethylene succinate) (PES) are aliphatic polyesters with similar chemical structures. The difference between the two polyesters is the number of diol units in the repeat units. PHS with more carbons per repeat unit not only has a faster crystallization rate and is more flexible but also has poor tensile strength and low flow melting point. In contrast, PES has a high melting point but a low elongation at break and a lower crystallization rate. Therefore, both polyesters have drawbacks and need to be modified to expand their application ranges. Yang et al. prepared poly(hexylene succinate-co-3 mol% ethylene succinate) (poly(HS-co-3 mol% ES)) copolyesters and studied their crystallization kinetics and melting behaviors(Yang and Qiu, 2013). They concluded that the two prepared P(HS-co-3 mol% ES) copolymers had the same characteristics as the pure PHS polymer, and the small number of ES units were in the amorphous regions of the copolyester, not in the crystalline PHS regions.

Few studies have considered the degradation behaviors of poly(hexamethylene succinate-co-ethylene succinate) (P(HS-co-ES)) copolyesters and associated changes in the physical properties of the polymer films before and after degradation. In this study, pure PHS, pure PES, and P(HS-co-ES) copolyesters with varying ES contents were synthesized using a two-step esterification and polycondensation reaction. The effects of the hydroxyl content on the physical properties of the resulting polyesters, including the crystal structure, thermal properties, and mechanical properties, were investigated. In addition, the polyesters were enzymatically degraded using cutinase, and the effects of polymer composition on the enzymatic hydrolysis rate are discussed. Moreover, the difference in film morphology, crystallinity, and thermal properties before and after enzymatic hydrolysis are compared.

## Material and Methods

### Material

Succinic acid (SA, 99.5%) and titanium isopropoxide (TTIP, 95%) were obtained from Shanghai Aladdin Biochemical Company (Shanghai, China). Ethylene glycol (EG, 98%), 1,6-hexanediol (HD, 98%), and decahydronaphthalene were purchased from Chengdu Aikeda Chemical Reagent Co., Ltd. (Chengdu, China). Chloroform was obtained from Shenyang Xinxing Reagent Factory (Shenyang, China). Anhydrous methanol was obtained from Tianjin Damao Chemical Reagent Factory (Tianjin, China). Cutinase was prepared following the procedure reported in our previous work (Hu et al., 2016). All chemicals and reagents were of analytical grade.

### Synthesis of Polyesters and Preparation of Samples

The molar ratio of SA to total diol(s) was 1:1.1. The reagents, including SA, EG, and HD, were added to 60 ml decahydronaphthalene containing TTIP at a mass concentration of 1/600 of the total reactant mass. The esterification reaction was carried out at 140°C for 2 h under a nitrogen atmosphere, and subsequently, the polycondensation reaction was carried out at 230°C for 4 h below 3 mmHg. The products were dissolved in 100 ml of chloroform and then precipitated into three times the volume of pre-cooled methanol. The precipitate was then washed with alcohol until the solution was clear, collected, and dried at 37°C under vacuum before use. The feed ratios of the diols used to prepare the different polymers are listed in Table 1.

**TABLE 1:** Composition and mechanical and thermal properties of P(HS-*co*-ES) copolyesters.

Polyester	HD/EG	HS/ES	Tensile strength/MPa	Elongation at break/%	Young’s modulus/MPa	T_m_/^o^C	X_c_/%
PHS	100/0	100/0	15.9 ± 1.9	52.5 ± 0.8	160.0 ± 2.2	55.5	50.9
P(HS-*co*-ES13)	80/20	87/13	13.7 ± 1.4	363.5 ± 6.4	49.8 ± 1.7	42.2	45.3
P(HS-*co*-ES32)	60/40	68/32	2.8 ± 0.6	66.0 ± 3.3	15.4 ± 0.5	30.6	39.5
P(HS-*co*-ES51)	40/60	49/51	0.4 ± 0.1	53.1 ± 1.8	3.0 ± 0.3	-	20.7
P(HS-*co*-ES76)	20/80	24/76	6.0 ± 0.4	7.7 ± 0.6	76.6 ± 2.6	58.2	49.8
PES	0/100	0/100	30.1 ± 1.7	8.5 ± 0.3	193.2 ± 4.7	104.1	58.3

The prepared polyesters were hot-pressed at 160°C and then cold-pressed at room temperature to obtain films with thicknesses of 1 and 0.5 mm, respectively. The 1-mm films were cut into 40 × 4 × 1 mm dumbbell-shaped pieces with a mold for the mechanical property tests. The 0.5-mm polyester films were cut into 30 × 10 × 0.5 mm rectangular pieces for the enzymatic hydrolysis and swelling experiments.

### Enzymatic Hydrolysis

The polyester films (30 × 10 × 0.5 mm) were vacuum-dried until their weight was kept constant, weighed, and then placed in 10 ml of Na_2_HPO_4_-NaH_2_PO_4_ buffer (0.1 M, pH = 7.4) containing 0.096 mg/ml cutinase at 37°C. The polyester films were removed from the buffer at regular intervals, rinsed with distilled water, dried with a clean absorbent wipe, and weighed. The removed films were dried under vacuum until the mass was constant to ensure that the water was removed and re-weighed. The weight loss ratios were calculated according to Eq. 1:

$$R=\frac{W_{0}-W_{d}}{W_{0}}\times 100\%,$$ (1)

where *R* is the weight loss ratio of the films; *W*_*0*_ is the initial film weight before the hydrolysis experiments, and *W*_*d*_ is the film weight after the films were incubated with the enzyme solution.

### Proton Nuclear Magnetic Resonance

The compositions of the polyesters were analyzed using 1H NMR spectroscopy (Bruker BioSpin, AVANCE III HD 400, Switzerland). Deuterated chloroform (CDCl_3_) was used as the solvent, and tetramethylsilane (TMS) was used as the internal standard.

### Attenuated Total Reflectance Fourier Transform Spectroscopy

ATR-FTIR data were collected using the ATR mode of an FT-IR spectrometer (Agilent Cary 660, United States of America) with a slide-on ATR accessory (Agilent, United States of America). The reported spectra are the average of sixteen scans that were collected over a frequency range from 4,000 to 400 cm^−1^ at a resolution of 2 cm^−1^.

### X-Ray Diffraction

The crystal structures of the polyester films were determined by XRD (S8 Tiger, Bruker, Germany). The XRD was equipped with Cu–Kα radiation source (*λ* = 0.1541 nm, 40 kV, 40 mA), and data were collected at 25°C. The scattering data were collected over a range of diffraction angles from 5 to 50° using a 0.02° step size. The crystallite sizes were evaluated from the XRD patterns according to the Debye–Scherrer Equation (Klug and Alexander, 1974).

### Differential Scanning Calorimeter)

The thermal properties of the polyester films were determined using DSC (TA Instruments, New Castle, DE, United States of America). The measurements were performed in a nitrogen atmosphere (50 ml/min). The polyester samples were heated from 0 to 150°C, held at 150°C for 3 min, cooled to 0°C, and then reheated to 150°C. The temperature ramp rate during all steps was 10°C/min.

### Thermogravimetry

The thermal decomposition behaviors of the polyester films were studied using TG analysis (TA Instruments, Q600, United States of America). About 8 mg samples were heated from room temperature to 500°C at a rate of 10°C/min under a nitrogen atmosphere (50 ml/min).

### Scanning Electron Microscopy

The surface morphologies of the polyester films before and after enzymatic hydrolysis were observed by SEM (SV810, Hitachi, Tokyo, Japan). For imaging, the film surfaces were sprayed with gold, and the films were placed on the sample stage for observation at 20 kV.

### Water Contact Angle Assay

The hydrophilicity of the dried polyester films surfaces was quantified by WCA measurements (KRUSS, DSA100, Hamburg, Germany). Static WCA angles were measured with an injection volume of 0.3 μL at 0.5 μl/s. The WCA values are reported as the average of five measurements at room temperature (25°C).

### Swelling Property Analysis

The polyester films (30 × 10 × 0.5 mm) were vacuum-dried to a constant weight, weighed, and the dimensions were measured and recorded. The polyester films were immersed in 50 ml of deionized water at room temperature. After 24 h of immersion, the films were removed from water, wiped dry, weighed, and the dimensions were re-measured. The swelling degree, Sw, of the polyester films was calculated according to Eq. 2:

$$S_{W}=\frac{W_{s}-W_{d}}{W_{d}}\times 100\%,$$ (2)

and the swelling ratios (Sr) were determined by Eq. 3:

$$S_{r}=\frac{A_{d}}{A_{s}},$$ (3)

where *W*_*d*_ is the dry wet weight of the polyester films, *W*_*s*_ is the weight of the film after it was immersed in water for 24 h, A_d_ is the side length of the dry polyester film, and A_s_ is the side length of the polyester film after it was swollen with water.

### Mechanical Property

The mechanical properties of samples were analyzed using an Instron 5500R universal testing machine (Instron Corp., Canton, Massachusetts, United States of America). The measurements were performed at room temperature with a tensile speed of 20 mm/min. The mechanical testing was carried out five times.

## Results and Discussion

### Composition of P(HS-co-ES) Copolyesters

**FIGURE 1:**
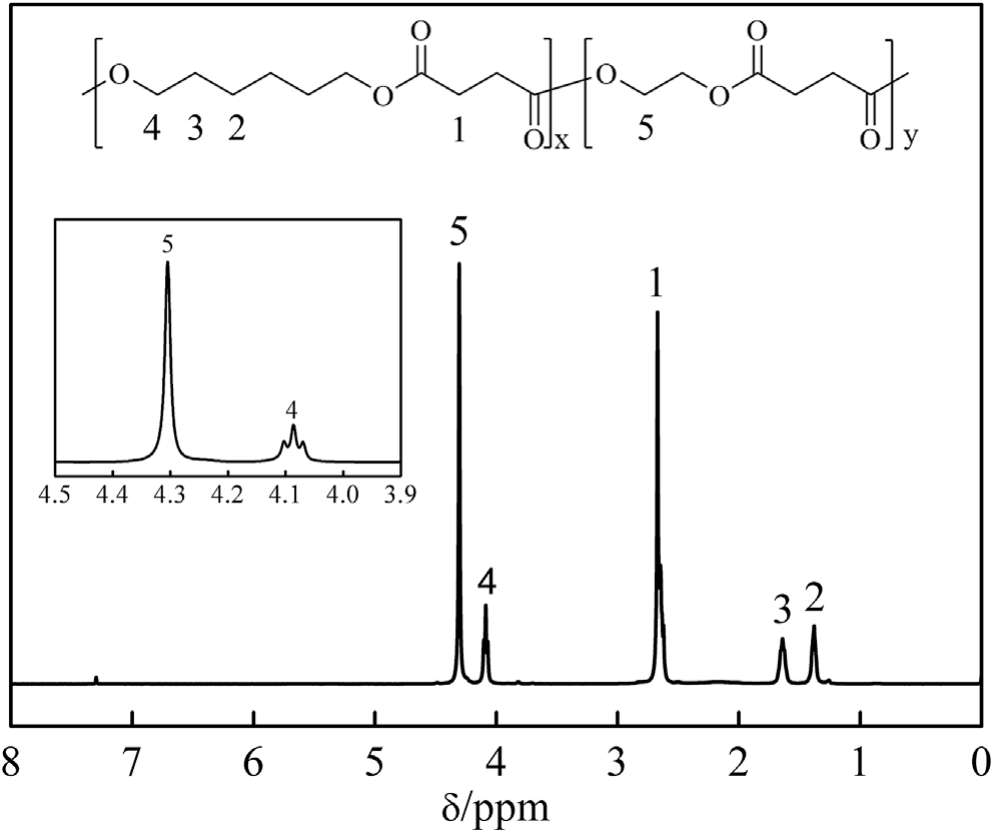
Chemical structure and ^1^H NMR spectrum of the prepared P(HS-*co*-ES76) copolyester.

The compositions of the synthesized P(HS-co-ES) copolyesters were determined by ^1^H NMR, and the measured ^1^H NMR spectrum and chemical structure for P(HS-co-ES76) are shown in Figure 1. The peak at 2.67 ppm is due to the protons on the COCH_2_ group in SA, labeled as 1 in the corresponding chemical structure. The peaks at 1.38, 1.64, and 4.09 ppm are assigned to the protons labeled 2, 3, and 4 in the HS units, respectively, and the peak at 4.30 ppm is assigned to proton 5 in the ES units. The ratios of the area of peak 4 from -O(CH_2_)_6_O- in the HS units to the area of peak 5 from -O(CH_2_)_2_O- in the ES units were used to calculate the molar ratio of HS/ES in the synthesized polymers (Tan et al., 2017; Bi et al., 2018). Polymers 10, 90. doi: 10.3390/polym10010090). The experimentally determined HE/ES ratios are expressed as mol%, and the deviation from the theoretical ratios is due to the volatilization of the ethylene glycol monomer during the reaction.

### Chemical Structure of P(HS-co-ES) Copolyesters

**FIGURE 2:**
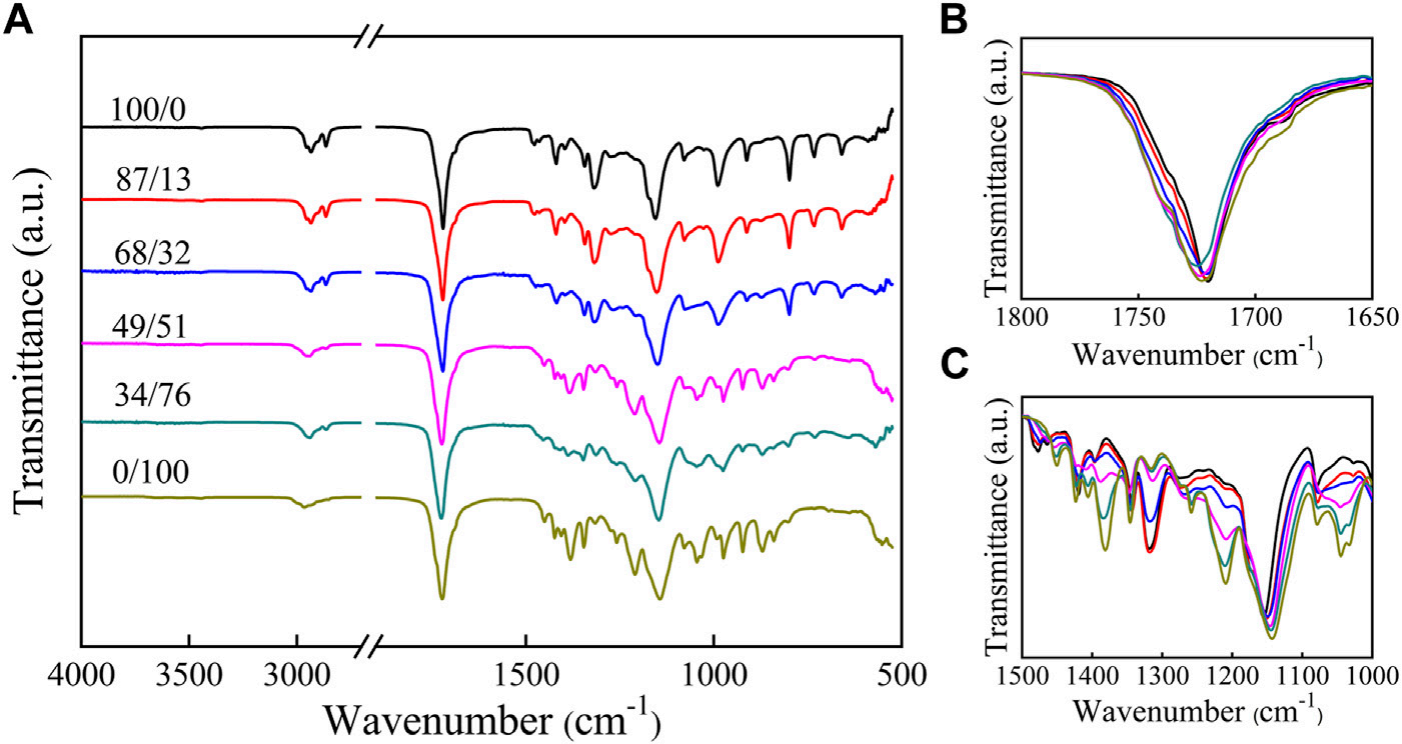
FT-IR spectra of PHS, PES, and P(HS-co-ES) copolyesters at various frequencies: **(A)** 4,000–500 cm^−1^; **(B)** 1800–1,650 cm^−1^; and **(C)** 1,500–1,000 cm^−1^.

FT-IR spectra of all synthetic polyesters are shown in Figure 2. The peaks at 2,930 and 2,860 cm^−1^ are attributed to -CH_2_- stretching vibrations. The peaks at 1720 cm^−1^ are attributed to the C=O stretching vibrations in the crystalline regions of the copolymers (Sato et al., 2004) and shifted to lower wavenumbers by approximatively 25 cm^−1^ compared to the unconstrained C=O groups due to the regular packing arrangement in the crystalline regions of the films(Sato et al., 2005). The band at 1,150 cm^−1^ is attributed to the C–O vibrations in the crystalline or amorphous regions of the films (Sato et al., 2004; Sato et al., 2005). The small absorption peak near 720 cm^−1^ in the FT-IR spectra measured for the PHS, P(HS-co-ES13), and P(HS-co-ES32) is assigned to the rocking vibrations of the - -(CH_2_)_4_- structure in the dibasic hydroxyl groups in HS (Karayannidis et al., 2003). In contrast, the diols used to synthesize PES only contained one methylene group, and therefore, the polyesters containing higher molar ratios of ES content did not have an absorption peak at 720 cm^−1^ from these in-plane swinging vibrations. The absorption peaks between 1800 and 1,650 cm^−1^ in the spectra measured for the P(HS-co-ES) copolyesters were similar to those of the pure PHS and PES polymers (Figure 2B); however, the positions, intensities, and shapes of the absorption peaks between 1,500 and 1,000 cm^−1^ are different (Figure 2C). In particular, the peak positions and shapes measured for copolyesters with HS contents >51 mol% are similar to those of PHS, while the peaks of the majority of ES copolyesters are similar to those of pure PES.

### Crystal Structure of P(HS-co-ES) Copolyesters

**FIGURE 3:**
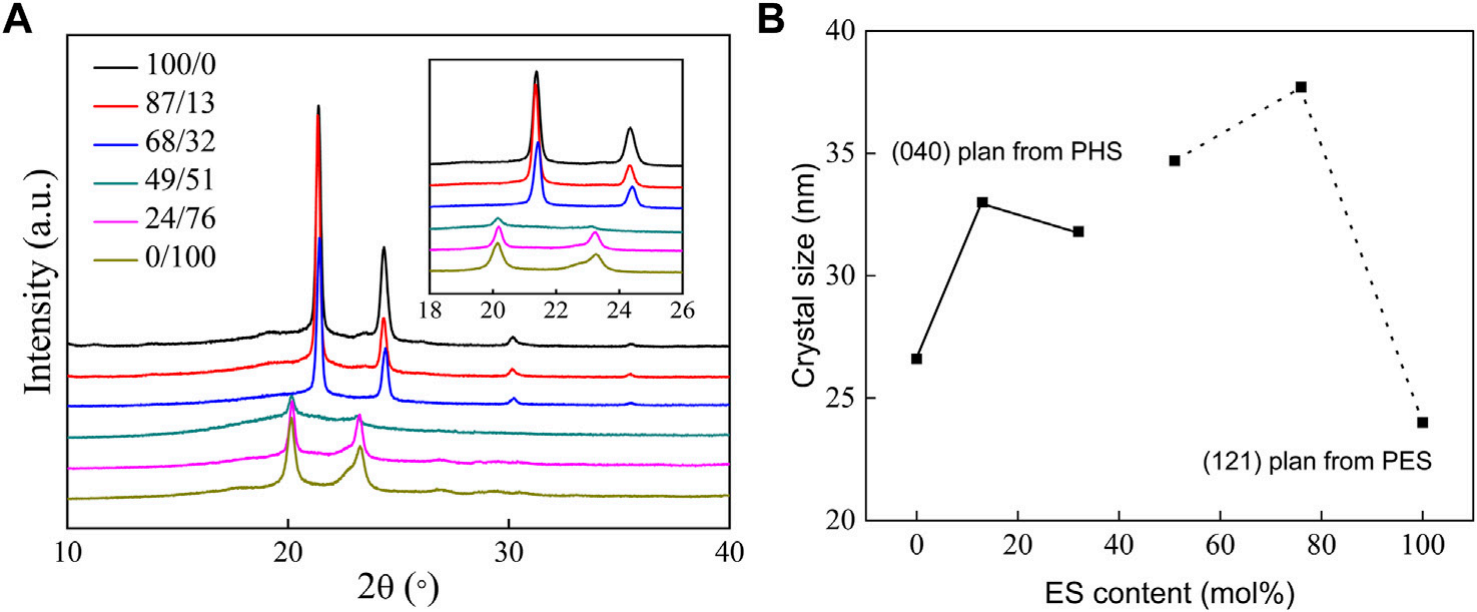
XRD patterns of PHS, PES, and P(HS-co-ES) copolyesters **(A)** and the corresponding crystallite sizes calculated using the Debye–Scherrer equation **(B)**.

The XRD diffraction patterns and crystal sizes of P(HS-co-ES) are shown in Figure 3. The data in Figure 3A show that PHS forms a monoclinic crystal with the diffraction peaks at 21.38°, 24.33°, and 30.22° that correspond to the (220), (040), and (240) planes, respectively. Meanwhile, PES is an orthorhombic crystal with diffraction peaks at 20.13°, 22.79°, and 23.22° from the (121), (200), and (220) planes, respectively (Bai et al., 2018a; Jing et al., 2021). The XRD diffraction patterns of copolyesters with ES contents of 13 mol% and 32 mol% are similar to those of PHS, indicating that ES was amorphous in these copolymers, while the XRD diffraction pattern of the copolyester with ES content greater than or equal to 51 mol% was similar to that of PES, indicating that the ES unit was crystalline in these copolyesters.

The degree of crystallinity (X_c_) in each sample was calculated from the areas of XRD diffraction peaks(Gigli et al., 2013), and the results are summarized in Table 1. The degree of crystallinity of all P(HS-co-ES) copolyesters is less than that of either the pure PHS or PES, regardless of the copolyester composition. The copolyester with an ES content of 51 mol% had the lowest degree of crystallinity among all the prepared polymers.

The crystallite sizes (L) were calculated from the half-width-half-max (HWHM) of the diffraction peaks using the Debye–Scherrer equation (Gigli et al., 2013). As shown in Figure 3B, the crystallite size of the (040) plane first increased and then decreased in the copolyesters compared to PHS, and the crystallite size of the (121) plane increased with increasing ES content. The crystallite sizes in the copolyester films are larger than those of pure PHS and PES, and the largest crystallite size was observed in the copolyester with an ES content of 76 mol% (Jing et al., 2021).

The melting temperatures (T_m_) determined from the second heating curves of the DSC measurements are listed in Table 1. No melting peaks were observed in the copolyester with an ES content of 51 mol% due to the low crystallinity of P(HS-co-ES51). The T_m_ of the copolyesters was lower than that of PHS and PES because introducing ES units into the copolyesters increases the number of methylene groups between the ester bonds, which reduces the chain order and packing (Nikolic and Djonlagic, 2001; Shi et al., 2019). The melting temperature also depends on the crystallite sizes in the polyester and the number of defects in the films, where the films with smaller crystals or more defects have lower T_m_ (Castilla-Cortázar et al., 2012). The DSC results revealed that P(HS-co-ES32) and P(HS-co-ES51) have low T_m_ values, while the XRD results showed that these samples had large crystal sizes, suggesting that the films formed by these two copolyesters had more crystal defects.

### Mechanical Properties of P(HS-co-ES) Copolyesters

The mechanical properties of the polyesters are summarized in Table 1. Although the tensile strengths for the different polyesters were similar, the elongations at break varied with the copolyester composition and first increased and then decreased with an increase in the ES content. The P(HS-co-ES13) copolyester had the highest elongation at break of 363.5%, while the P(HS-co-ES76) copolyester had the lowest elongation at break of 7.7%, which was also lower than that of either the pure PHS or PES polyesters. Furthermore, comparison of the properties of the different polyesters reveals a correlation between the mechanical properties and thermal and crystal properties. The melting temperature and crystallinity of the copolyesters are lower than those of PHS and PES, while the elongations at break are higher, which may be because the degree of crystallinity decreases with increasing ES content, and the corresponding increase in the size of the amorphous regions of the films leads to a decrease in the stiffness. Meanwhile, the tensile strength and Young’s modulus of P(HS-co-ES32) and P(hs-co-ES51) are very low possibly because of their low degrees of crystallinity and melting temperatures as the soft amorphous phase leads to more defects in the films and makes the copolyesters softer and less viscous at room temperature. The change in the tensile properties of P(HS-co-ES) is consistent with other reported results (Zhang et al., 2021).

### Enzymatic Hydrolysis of P(HS-co-ES) Copolyesters

**FIGURE 4:**
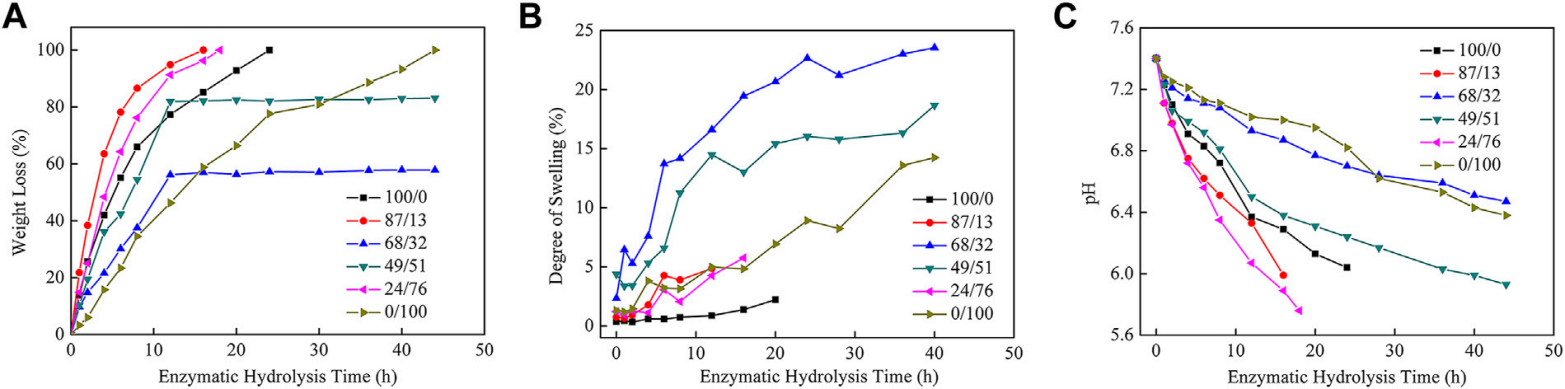
Weight loss curves **(A)**, degree of swelling curves **(B),** and pH curves **(C)** of polyester films after hydrolysis by cutinase.

Figure 4A shows the weight loss of the hydrolyzed polyester films after they were incubated with cutinase at pH 7.4 and 37°C. There was no measurable weight loss in the control samples incubated in the same buffer without cutinase (data not shown). The P(HS-co-ES32) and P(HS-co-ES51) samples (Figure 4A) were not completely hydrolyzed during the experiments because the films curled as they degraded. The total surface area decreased during the curling process; therefore, the weight loss of the P(HS251-co-ES32) and P(HS-co-ES51) was less.

Figure 4B shows that the amount of water absorbed by the polyesters increased throughout the enzymatic hydrolysis process, where P(HS-co-ES32) adsorbed the most water absorption, followed by P(HS-co-ES52). The degree of swelling of the other four polyesters did not exceed 15%, and PHS absorbed the least water. After 4 h of incubation with the enzyme solution, the enzymatic hydrolysis rates of PHS, P(HS-co-ES13), P(HS-co-ES32), P(HS-co-ES51), P(HS-co-ES76), and PES were 42.03, 63.50, 21.61, 36.10, 48.37, and 15.74%, respectively. In addition, it took about 5, 3, 10, 8, 5, and 14 h to hydrolyze 50% of the mass of each polyester. The addition of ES significantly increased the rate of enzymatic hydrolysis of the copolyesters, with the exception of P(HS-co-ES32) and P(HS-co-ES51). P(HS-co-ES13) and P(HS-co-ES76) completely hydrolyzed in less time than pure PHS and PES, and P(HS-co-ES13) has the highest enzymatic hydrolysis rate under the same experimental conditions.

P(HS-co-ES13) has the fastest enzymatic hydrolysis rate. In the first, rapid degradation stage, the ester bonds were degraded, resulting in shorter polyester segments and significant weight loss. In the second, slower degradation stage, the terminal fragments were enzymatically hydrolyzed, forming water-soluble oligomers that dissolved away from the film surface (Rizzarelli et al., 2004; Bikiaris et al., 2006). The pH of the surrounding buffer solution decreases as the ester bonds are cleaved and water-soluble substances are produced (Figure 4C), and cutinase is less active at lower pHs, which leads to a slowing down of the enzymatic hydrolysis rate. The total surface area of the P(HS-co-ES32) and P(HS-co-ES51) samples decreased when the films curled, and as a result, these samples did not completely hydrolyze. Overall, the enzymatic hydrolysis rates of these samples were relatively slow, especially in the second stage, when the enzymatic hydrolysis rate was almost zero. Except for P(HS-co-ES32) and P(HS-co-ES51), the enzymatic degradation rate of the other polyesters followed P(HS-co-ES13) > P(HS-co-ES76) > PHS > PES. The main factors affecting enzymatic hydrolysis are hydrophilicity, melting temperature, and crystallinity, each of which is discussed in the next sections.

**FIGURE 5:**
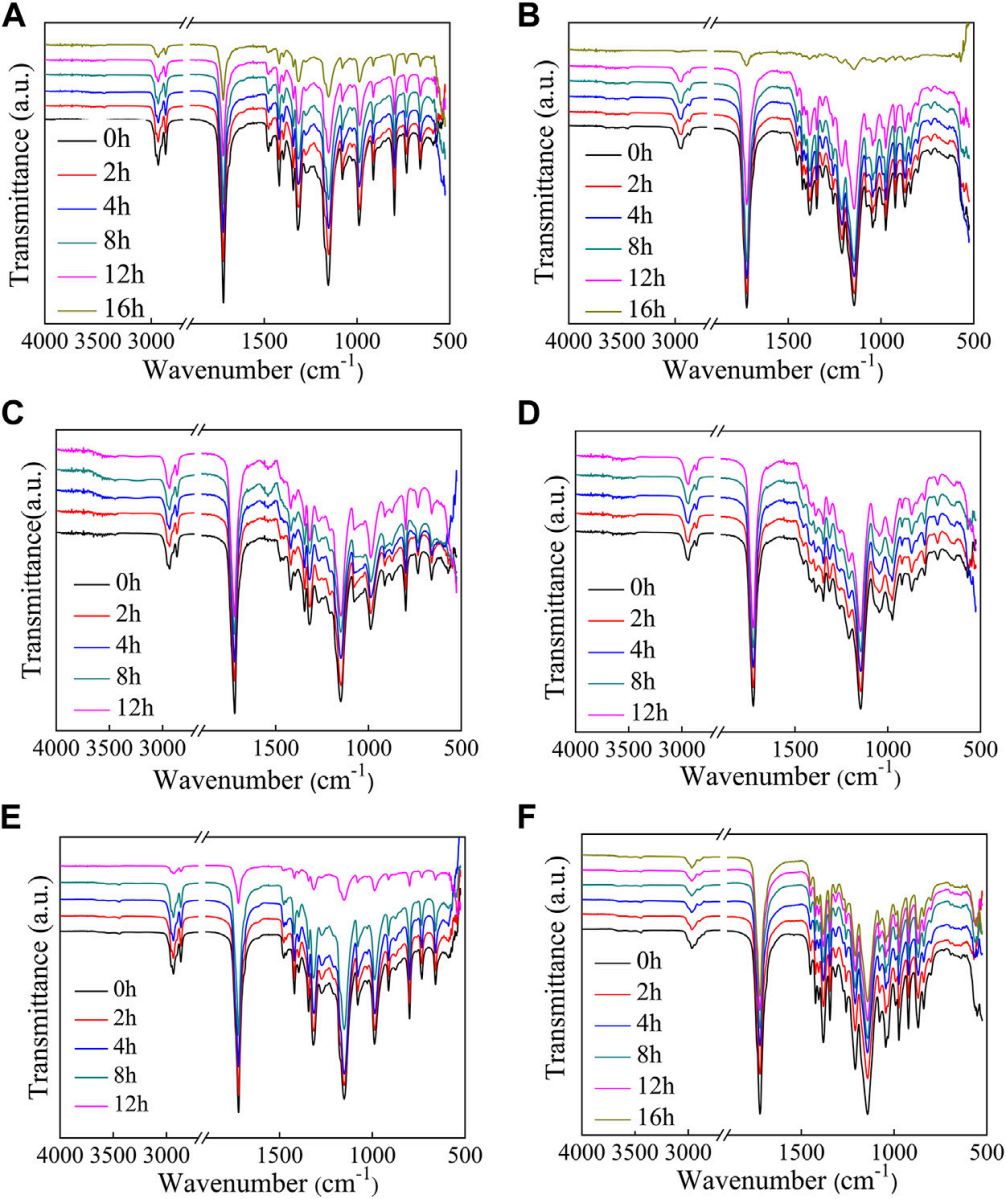
FT-IR spectra measured of the polyesters at different extents of enzymatic hydrolysis. **(A–F)**: PHS, P(HS-co-13ES), P(HS-co-32ES), P(HS-co-51ES), P(HS-co-76ES), and PES.

The FT-IR spectra of polyesters after enzymatic hydrolysis are compared in Figure 5A–F. The peak positions did not change significantly; however, the intensity of the C=O (1720 cm^−1^) and C–O (1,150 cm^−1^) absorption peaks decreases with an increase in the hydrolysis time. The decrease in peak intensity is due to the pores that formed as the films degraded.

**FIGURE 6:**
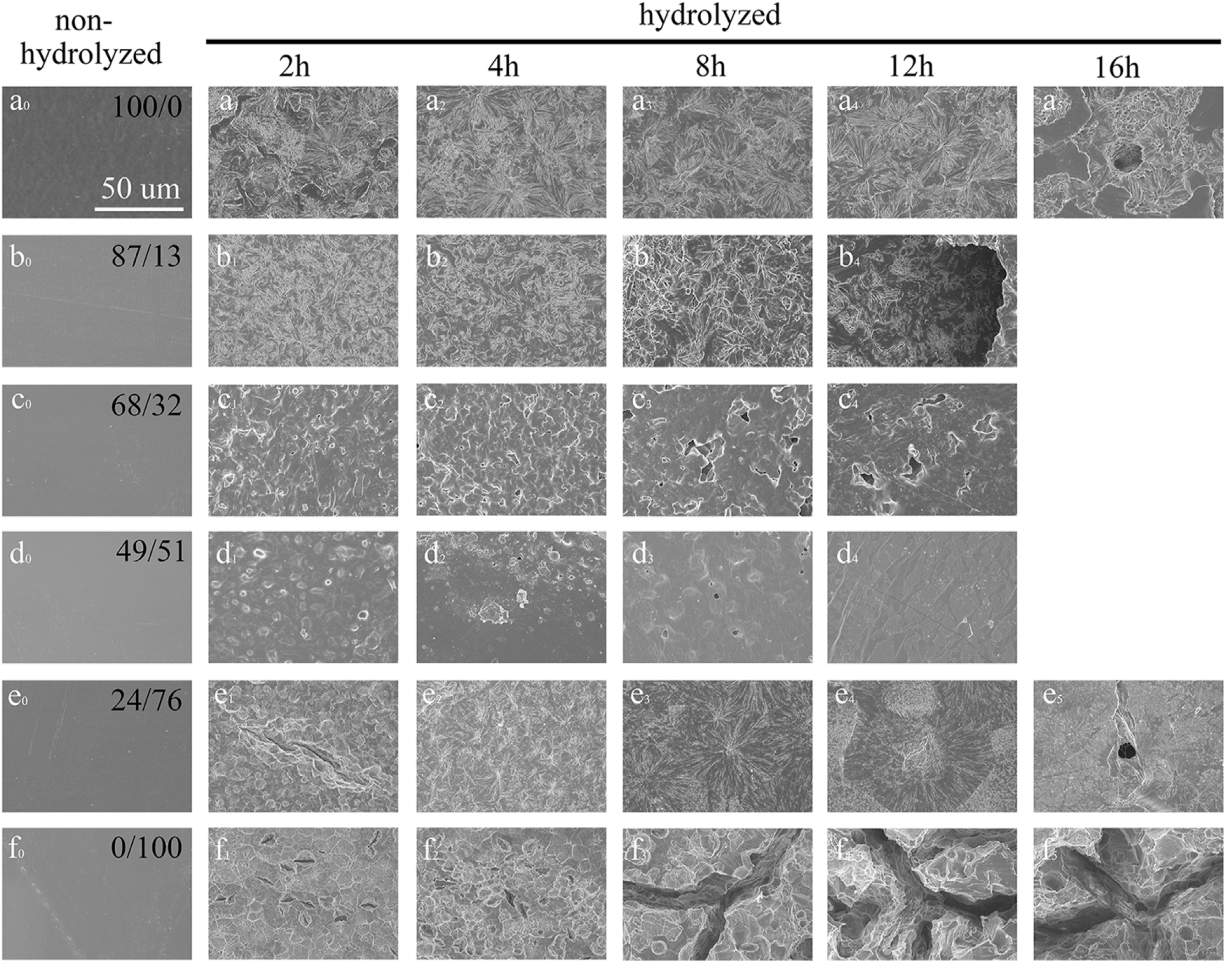
SEM images of the polyester films after enzymatic hydrolysis with cutinase. **(A–F)**: PHS, P(HS-*co*-13ES), P(HS-*co*-32ES), P(HS-*co*-51ES), P(HS-*co*-76ES), and PES.

**FIGURE 7:**
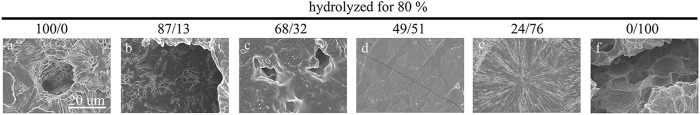
SEM images of the polyester films after 80% of their masses were enzymatically hydrolyzed. **(A–F)**: PHS, P(HS-*co*-13ES), P(HS-*co*-32ES), P(HS-*co*-51ES), P(HS-*co*-76ES), and PES).

Figure 6 and Figure 7 show the surface morphology of the polyester films after enzymatic hydrolysis. Before enzymatic hydrolysis, the polyester films had smooth surfaces (Figures 6a_0_-f0). The surface morphologies of neat PHS and PES films are rough and nonuniform after 2 h of incubation with the cutinase solution, and pits and cracks appear with an increase in the hydrolysis time as more of the ester bonds are cleaved. At the end stages of hydrolysis, the pits are deep and large (Figures 6a_5_/f_5_) due to the penetration of water into the amorphous regions of the films, which in turn increases the rate of enzymatic hydrolysis (Azevedo et al., 2003). The results show that enzymatic hydrolysis occurs on the surface of the polyester films. The surface morphologies of P(HS-co-ES13) and P(HS-co-ES76) films are similar to those of the neat PHS and PES films, respectively. Meanwhile, the surface morphology of the P(HS-co-ES32) and P(HS-co-ES51) films did not change significantly after enzymatic hydrolysis. The surface of the P(HS-co-ES51) film remains relatively smooth after 12 h with only a few cracks due to incomplete enzymatic hydrolysis. These copolyesters are molten during the enzymatic hydrolysis experiments at 37°C and then recrystallized at room temperature for imaging, leading to formation of some cracks. Figure 7 compares the surface morphologies of the copolyester films after 80% of their mass is lost, except for the image of the P(HS-co-ES51) film, where the degree of degradation was 50%. The water molecules easily penetrate the pits and cracks on the surface of the films, which leads to further corrosion and increases the rate of weight loss.

**FIGURE 8:**
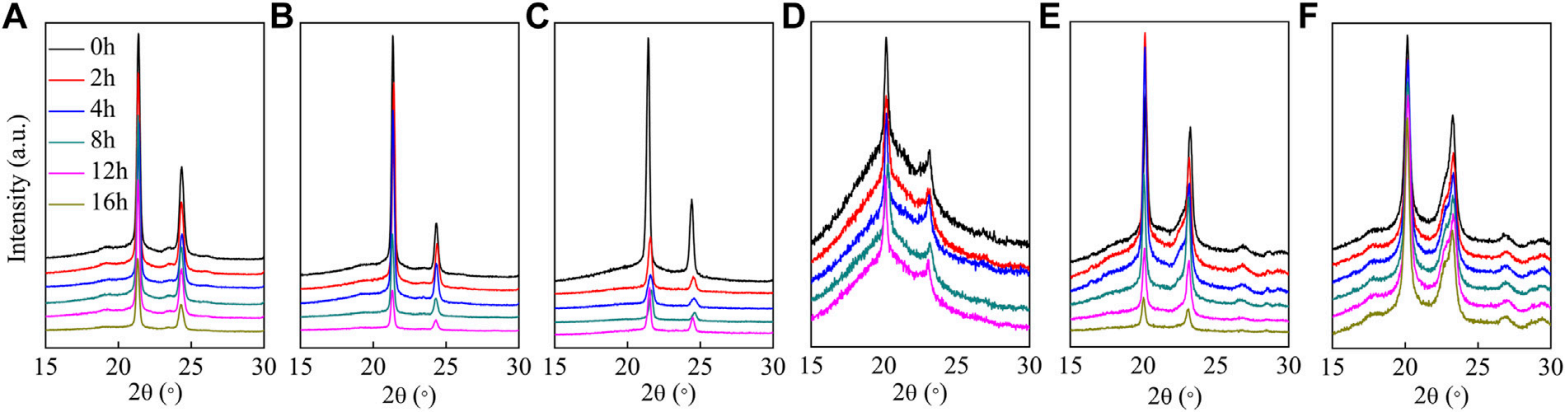
XRD patterns of the polyesters at different extents of enzymatic hydrolysis. **(A–F)**: PHS, P(HS-*co*-13ES), P(HS-*co*-32ES), P(HS-*co*-51ES), P(HS-*co*-76ES), and PES.

XRD patterns and the corresponding X_c_ of the hydrolyzed polyesters are presented in Figure 8; Table 2, respectively. As shown in Figure 8A–F, the positions of diffraction peaks did not change after enzymatic hydrolysis, indicating that the crystal structure did not change(Bai et al., 2018b); however, the degree of crystallinity varied with the copolyester chemical composition. The degree of crystallinity impacts the rate of enzymatic hydrolysis of the polyester (Bai et al., 2018a), where the materials with higher degrees of crystallinity have slower enzymatic hydrolysis rates (Seretoudi et al., 2002; Yasuniwa and Satou, 2002; Bikiaris et al., 2006). Although P(HS-co-ES51) has the lowest crystallinity (about 20.7%), the film was in a molten state during the hydrolysis experiments and did not completely hydrolyze at 37°C, suggesting that low degrees of crystallinity may also prevent the films from solidifying in the hydrolysis experiment conditions. Similarly, P(HS-co-ES32) eventually became molten during the long hydrolysis experiments, which causes the surface area to decrease and results in incomplete hydrolysis. Yet, P(HS-co-ES32) and P(HS-co-ES51) also have a greater hydrolysis rate in the earlier period. The degree of crystallinity of the prepared polyesters increases as P(HS-co-ES13) < P(HS-co-ES76) < PHS < PES, and the enzymatic hydrolysis rate follows the exact opposite trend, with P(HS-co-ES13) > P(HS-co-ES76) > PHS > PES, emphasizing that the polyester films with lower degrees of crystallinity degrade faster (Marten et al., 2003; Abou-Zeid et al., 2004; Zhang et al., 2021).

**TABLE 2:** Thermal properties of the polyesters after enzymatic hydrolysis.

Polyester	Time (h)	T_m_ (^o^C)	X_c_ (%)	T_5%_ (^o^C)
PHS	0	59.9	50.9	321
	2	59.6	49.8	317
	4	55.9	50.1	315
	8	61.6	50.6	313
	12	56.2	47.6	303
	16	60.0	45.8	308
P(HS-*co*-ES13)	0	55.3	45.3	330
	2	51.9	43.8	321
	4	45.5	44.5	326
	8	50.1	42.2	327
	12	51.7	41.3	325
P(HS-*co*-ES32)	0	44.9	39.5	343
	2	46.6	38.8	318
	4	46.7	39.1	327
	8	43.9	37.6	323
	12	32.7	33.7	315
P(HS-*co*-ES51)	0	-	20.7	333
	2	-	20.1	328
	4	-	19.9	299
	8	-	18.9	318
	12	-	18.4	254
P(HS-*co*-ES76)	0	71.8	49.8	322
	2	69.7	48.2	397
	4	69.8	48.6	307
	8	70.0	49.5	309
	12	70.4	49.4	309
	16	-	48.2	309
PES	0	103.9	58.3	303
	2	102.2	57.8	302
	4	101.9	56.4	302
	8	102.1	56.2	301
	12	102.1	55.9	297
	16	102.6	56.6	293

The cutinase used in this work was expressed with the gene from *Fusarium solani* and had excellent degradation performance as it was able to penetrate and hydrolyze the polyesters (Bai et al., 2018a). As the hydrolysis time increases, the area of the diffraction peaks in the XRD pattern measured of the hydrolyzed films has no obvious changes, but some of them decrease at the end of hydrolysis, indicating a decrease in X_c_ (Table 2). At the end of hydrolysis, the low molecular degradation products and the increased water absorption in the film decrease the degree of crystallinity (Sato et al., 2004).

The melting temperatures of polyester films also impact their rate of enzymatic hydrolysis (Bai et al., 2018a). Table 2 lists the melting temperature of the polyesters before and after enzymatic hydrolysis determined from the first DSC heating curve. Because P(HS-co-ES32) and P(HS-co-ES51) became molten during the enzymatic hydrolysis process at 37°C, the total surface area of the films became smaller, which affected their enzymatic hydrolysis rate. The melting temperature of the P(HS-co-ES13) copolyester is lower than that of the neat PHS and PES, and as expected, the enzymatic hydrolysis rate of this film was faster than either of the neat polyester films. In addition, there is no significant difference in T_m_ before and after hydrolysis. Moreover, only P(HS-co-ES76) has a higher melting temperature than neat PHS, yet it hydrolyzes faster than neat PHS, indicating that there must be other factors that can influence the rate of enzymatic hydrolysis.

The thermal decomposition temperature (T_5%_) at an initial mass loss of 5% is listed in Table 2 for the prepared polyesters. It is to be noted that the T_5%_ values of the P(HS-co-ES) copolyesters are higher than those of either of the pure polymers. As the extent of the enzymatic hydrolysis increases, the thermal decomposition temperature decreases because more end groups form as the polymers degrade, which further facilitates the thermal decomposition of the polyesters (Bikiaris and Karayannidis, 1998). As the macromolecular chains degrade into small molecular oligomers and fragments, the number of carboxyl and hydroxyl end groups increases (Bikiaris and Karayannidis, 1999). Therefore, the thermal decomposition temperature of the polyester decreases, but this decrease is not significant, particular for P(HS-co-ES13), where the temperature decreases by less than 10°C.

The surface wettability of the polyester films was evaluated by measuring the water contact angle, and the results are listed in Table 3. The water contact angles of the P(HS-co-ES) copolyester films are smaller than those of PHS and PES, indicating that copolyesters films are more hydrophilic than either of the neat PHS and PES films. In particular, the P(HS-co-ES32) and P(HS-co-ES51) copolyester films have lower water contact angles and are more hydrophilic, while the P(HS-co-ES13) and P(HS-co-ES76) copolyester films have higher water contact angles and were more hydrophobic. It should be noted that the wettability only reflects the hydrophilicity of the surface of the polyester films and is not directly related to the hydrophilicity of polyesters (Zeng et al., 2011). The experimental results show that the surface of the copolyester films was more wettable.

**TABLE 3:** Water contact angle, degree of swelling, and swelling ratio of the polyesters.

Polyester	WAC (^ο^)	S_w_ (%)	S_r_
PHS	94.7	1.071	1.005
P(HS*-co*-ES13)	93.3	1.195	1.004
P(HS*-co*-ES32)	88.6	0.715	1.005
P(HS-*co*-ES51)	89.3	0.265	1.002
P(HS-*co*-ES76)	94.3	0.129	1.002
PES	95.5	0.006	1.002

The swelling properties of the polyester films are related to the amount of water absorbed by the films to some extent (Lee et al., 2013). It can be seen from Table 3 that the swelling ratio of the polyesters did not change after the films were degraded; however, the degree of swelling did change with the ES content in the copolyester and is higher than that of pure PES. The largest degree of swelling was measured for the copolyester with an ES content of 13 mol%.

Based on the water contact angles, the hydrophilicity of polyesters follows P(HS-co-ES13) > P(HS-co-ES76) > PHS > PES, the same trend as the enzymatic hydrolysis rate. In other words, the most hydrophilic polymers have the fastest degradation. In summary, the main factors affecting the rate of enzymatic hydrolysis are hydrophilicity, melting temperature, and crystallinity, which can be tuned by adjusting the composition of the hydroxyl monomers in the copolyesters.

## Conclusion

In this work, a series of P(HS-co-ES) copolyesters containing different HS/ES ratios were synthesized by adding HD/EG to improve the performance of the pure PHS and PES polyesters and expand their fields of application. The research results show that the mechanical properties of the P(HS-co-ES) copolyesters improved compared to the pure polyesters. The crystal structures of the copolyesters were similar to those of the corresponding pure polymer which was the majority monomer unit in the copolymer. The thermal decomposition temperatures of all polyesters were higher than 290°C and met the temperature requirements for industrial production. The enzymatic hydrolysis rates of the copolyester films using cutinase were faster than those of the pure polyesters. The physical and biodegradability properties of the polyesters could be tuned by adjusting the composition of the hydroxyl monomers in the copolyesters, where polyesters that were more hydrophilic had lower melting temperatures, lower degrees of crystallinity, and biodegraded faster. Among the prepared copolyesters, P(HS-co-ES13) was a soft but tough polymer with a high hydrolysis rate, while P(HS-co-ES76) was a partially brittle copolymer that also had a high hydrolysis rate. The tunable properties show that the copolyesters with different compositions can be applied in different fields.

## Data Availability

The raw data supporting the conclusion of this article will be made available by the authors, without undue reservation.
